# Asymmetric dual-core liquid crystal channel-based tunable mode converter

**DOI:** 10.1038/s41598-024-55609-1

**Published:** 2024-03-04

**Authors:** Mohamed Saleh Mohamed Esmail, Mohamed Farhat O. Hameed, Salah S. A. Obayya, B. M. Younis

**Affiliations:** 1 Basic Science Department, Faculty of Engineering, Misr University for Science and Technology, Giza, 12588 Egypt; 2 Center for Nanotechnology, Zewail City of Science, Technology and Innovation, October Gardens, 6th of October City, Giza, 12578 Egypt; 3 Nanotechnology and Nanoelectronics Engineering Program, Zewail City of Science, Technology and Innovation, October Gardens, 6th of October City, Giza, 12578 Egypt; 4 Centre for Photonics and Smart Materials, Zewail City of Science, Technology and Innovation, October Gardens, 6th of October City, Giza, 12578 Egypt; 5 Department of Electronics and Communications Engineering, Misr Higher Institute for Engineering and Technology (MET), Mansoura, Egypt

**Keywords:** Engineering, Optics and photonics

## Abstract

In this work, a higher order-to-fundamental mode converter is reported and analyzed based on an asymmetric dual channel waveguide (ADC-WG) on silicon. In the reported structure, one of the two waveguides is infiltrated with nematic liquid crystal (NLC) material to add temperature tunability while the other one is a solid BK7 waveguide. The modal characteristics are obtained using the full vectorial finite difference method (FVFDM). In addition, the structural parameters and optical characteristics of the employed materials are investigated to achieve good wavelength selectivity with a short device length (L_D_). Thus, a compact mode converter that can work at different wavelengths including the telecommunication wavelength i.e., 1.55 μm with L_D_ ~ 482.31 μm and a low crosstalk of − 19.86 dB is presented. To prove the thermal tunability of the suggested mode converter, its operation is tested through a temperature range between 20 and 35 °C and the results show that the mode conversion process is achieved at each temperature with different phase matching wavelengths (λ_PMW_) but with quite similar coupling length (L_C_). The proposed device can therefore be effectively utilized in integrated photonic circuits.

## Introduction

Optical mode conversion has recently caught the interest of numerous researchers^1–3^. The process of transferring most of the power from one mode guided in a certain waveguide to another mode (usually of different order) supported by a different waveguide is referred to as optical mode conversion. This operation may enhance the data transfer rate and transmission capacities of optical networks^4,5^. With the use of a layered silicon-insulator-silicon substrate, silicon devices are made using the silicon-on-insulator (SOI) platform, which reduces parasitic capacitance within the device and improves performance^6^. SOI technology is the manufacture of silicon semiconductor devices in layered silicon–insulator–silicon substrate^7^. The silicon junction of SOI-based devices is located atop an electrical insulator, usually silicon dioxide or sapphire, setting them apart from ordinary silicon-built devices (these types of devices are called silicon on sapphire, or SOS). The application is a major factor in selecting an insulator. Thus, silicon dioxide is utilized in various microelectronic devices to reduce short-channel effects, while sapphire is utilized in high-performance radio frequency and radiation-sensitive applications^8,9^. Therefore, many active/passive photonic devices such as electro-optical (EO) modulators and optical sensors based on the SOI platform^10^, have been proposed. Beam splitters, demultiplexers, and mode converters are only few of the numerous uses for dual-core waveguides based on the SOI platform^11–13^. Since asymmetry between two waveguide cores results in significant benefits for the optical response, such as high birefringence and compactness, dual asymmetric core photonic devices have been widely used in a variety of applications^14,15^. In addition, photonic devices can be partially infiltrated with fluid materials such as polymers^16^, oil^17^, or liquid crystals (LCs)^18^ to handle the polarization characteristics of the guided modes. However, because LCs are anisotropic and can have their refractive indices changed by adjusting the temperature or applying an external electric field, they provide a superior substitute for conventional fluid materials^19^. The tunability of LC materials against both temperature and applied electric field makes the infiltration of the LCs through the SOI structures extremely advantageous^20,21^. To introduce various tunable photonic devices, such as filters^22^, polarization beam splitters^19,23^, optical switches^24^, sensors^25^, and mode converters^26^, LCs infiltrating photonic devices have been employed.

Intermodal coupling in asymmetric dual-core photonic crystal fibers (ADC-PCF) has recently been reported and studied in the context of mode conversion^27–30^. Lin et al.^27^ have introduced an LP_01_ to LP_02_ mode converter based on a unique DC-PCF for dispersion adjustment. With a bandwidth of 22 nm at λ = 1.55 μm, the reported device in^27^ achieves 80% coupling efficiency. Additionally, the simulation results revealed that the bandwidth is increased to 31 nm^27^ by tapering the dual-core PCF. By introducing an asymmetric DC-PCF, Chen et al.^28^ have first described the mode coupling between a small core and a larger one. Such a mode converter^28^ has a 12.7 mm device length and a 14 nm bandwidth at λ = 1.55 μm. Furthermore, Cai et al.^29^ have suggested a hybrid DC-PCF mode converter that consists of an index-guiding core and a photonic bandgap guiding one. In this study^29^, the bandwidth, phase-matching wavelength, and coupling efficiency can be controlled by adjusting the refractive index of the hole between the dual cores. Additionally, Yu et al.^30^ have shown that a tunable magnetic fluid-filled hybrid PCF mode converter can convert the LP_11_ mode in the index-guiding core to the LP_01_ mode in the photonic bandgap-guiding core. The coupling efficiency can approach 95% using a 835 μm long fiber^30^ in the wavelength range from 1380 to 1750 nm, with a phase-matching bandwidth of 370 nm. In addition, a tunable mode converter based on dual core PCF where one core is infiltrated with NLC has been proposed in^31^. In this study, the phase matching wavelength changes from 1.285 to 1.3634 µm by changing the temperature from 15 to 45 °C with a device length 0.4036 mm. While all the reported mode converters in literature have their own advantages, obtaining a tunable, compact, and easy to fabricate device is still a challenge.

In this work, the proposed mode converter depends on the asymmetry between two neighboring channel waveguides on a silicon substrate with SiO_2_ cladding layer. The coupling between the two cores is the fundamental concept of the suggested mode converter. This coupling is strong only at the phase-matching wavelength (λ_PMW_), which allows the phase-matching criterion to be attained^31^. To create a structure of dual asymmetric channels, in which the two waveguides do not have the same dimensions, one of the two channels is infiltrated with NLC material of type E7 and the other one is a solid BK7 waveguide. Asymmetry is employed to gain the necessary attitude at a specific wavelength that may be modified thanks to the NLC temperature tunability. Thus, the first higher-order mode supported by the launching (NLC) core is strongly coupled to the fundamental mode of the BK7 core. To produce the necessary tunable and switchable behavior, the NLC parameters i.e., rotation angle and temperature can be changed. The proposed mode converter has a short L_D_ of ~ 482.31 μm and can work efficiently at different wavelengths with a low crosstalk of − 19.86 dB. The suggested mode converter operates at a temperature range between 20 and 35 °C and the results show that the mode conversion process is attained with a similar coupling length at each temperature where the λ_PMW_ can be tuned via the NLC temperature. The characteristics of the studied modes are obtained using the full vectorial finite difference method (FVFDM) via the Lumerical software package^32^. In terms of the device length (L_D_), thermal tunability, and crosstalk, the operation of the reported mode converter outperforms that of similar devices^27–30^, recently presented in the literature.

### Design considerations and simulation methodology

**Figure 1:**
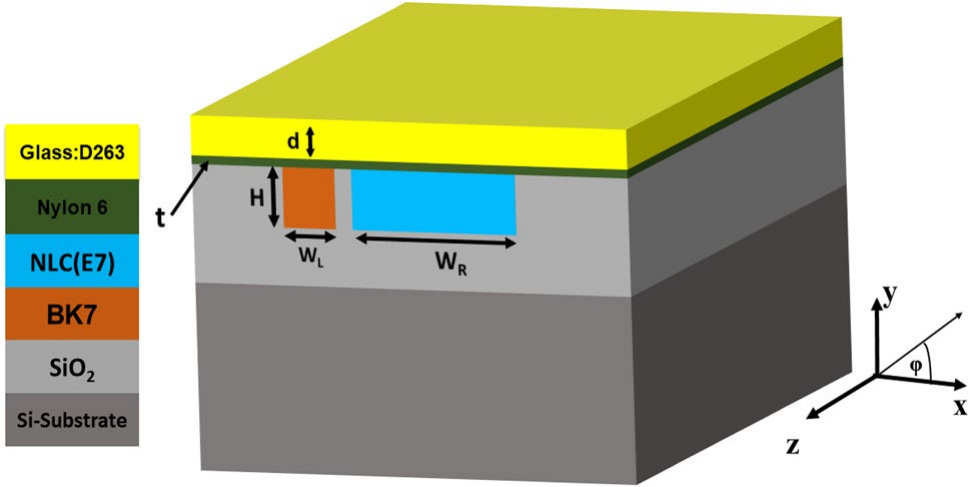
3D schematic of the dual-core waveguide-based proposed adjustable mode converter.

The suggested design is shown in Fig. 1. The proposed structure consists of two-channel waveguides on silicon (n = 3.5 at λ = 1.55 μm) while the cladding region is silica (SiO_2_) with a refractive index of 1.44 as may be seen in Fig. 1.

The right core region is etched in the SiO_2_ cladding and infiltrated with nematic liquid crystal (NLC) of type E7 while the neighboring (left) core is infiltrated with BK7 material to perform a dual asymmetric core structure. The NLC (right) core has a height (H) of 5 μm and a width (W_R_) of 9.8 μm. On the other hand, the BK7 (left) core has the same height (H) but with a different width (W_L_) of 5 μm to keep the structural asymmetry between the two cores. A very thin coating of Nylon 6 with a thickness of 50 nm and a refractive index of 1.5419 is placed over the common surface made up of SiO_2_, NLC, and BK7 materials^33^. Nylon 6 layer can be mechanically rubbed to obtain a uniform alignment of the NLC molecules in the desired direction. Nylon 6 is created as a thin photo-alignment layer on a glass plate to improve the planar, homogenous alignment of the NLC molecules^34^. Another layer of D263 glass with a thickness of 1.5 μm and a refractive index of 1.506 is placed on top of the thin Naylon 6 layer to enclose the NLC material and features a top cladding^35^. Both silica and D263 glass have a very low chromatic dispersion (dn/dλ)^35,36^ and equal to − 0.01256 μm^−1^ and − 0.0122 μm^−1^, respectively. Both materials have nearly constant refractive indices throughout the studied wavelength range i.e., 1.4–1.8 μm. So, the dispersion of silica and D263 is not considered in our study as it is expected to have no effect on the modal characteristics. The refractive index of the Nylon 6 (n = 1.5419) material is used as a single value as reported in similar research in literature^34^. In addition, the Nylon layer in the proposed work has a thickness of only 50 nm in size and is expected to have no effect on the modal characteristics of the presented mode converter. Nylon 6 layer can be mechanically rubbed to obtain a uniform alignment of the NLC molecules in the desired direction. Nylon 6 is created as a thin photo-alignment layer on a glass plate to improve the planar, homogenous alignment of the NLC molecules^34^. It is worth noting that the dispersions of the core materials i.e., BK7 and NLC are considered in the proposed study.

As an anisotropic material, the NLC is characterized by two refractive indices, the extraordinary refractive index (n_e_) and the ordinary refractive index (n_o_) while its dielectric permittivity tensor is depicted in Eq. (1)^37^:

1 $${\varepsilon }_{r}=\left[\begin{array}{lll}{n}_{o}^{2}{{\text{sin}}}^{2}\varphi +{n}_{e}^{2}{{\text{cos}}}^{2}\varphi &\quad \left({n}_{e}^{2}-{n}_{o}^{2}\right)sin\varphi cos\varphi &\quad 0\\ \left({n}_{e}^{2}-{n}_{o}^{2}\right)sin\varphi cos\varphi &\quad {n}_{o}^{2}{{\text{cos}}}^{2}\varphi +{n}_{e}^{2}{{\text{sin}}}^{2}\varphi &\quad 0\\ 0&\quad 0&\quad {n}_{o}^{2}\end{array}\right]$$

where $\varphi$ is the angle between the x-direction and the NLC director (inset of Fig. 1). The Cauchy equations that yield n_e_ and n_o_ are taken from^38^ and depicted as Eqs. (2) and (3).

2 $${n}_{e}={A}_{e}+\frac{{B}_{e}}{{\lambda }^{2}}+\frac{{C}_{e}}{{\lambda }^{4}}$$

3 $${n}_{o}={A}_{o}+\frac{{B}_{o}}{{\lambda }^{2}}+\frac{{C}_{o}}{{\lambda }^{4}}$$

where A_e_, B_e,_ C_e_, A_o_, B_o_, and C_o_ are the Cauchy coefficients. At temperature^38^ T = 25°C, A_e_ = 1.6933, B_e_ = 0.0078 μm^2^, C_e_ = 0.0028 μm^4^_,_ A_o_ = 1.4994, B_o_ = 0.007 μm^2^, and C_o_ = 0.0004 μm^4^.

**Figure 2:**
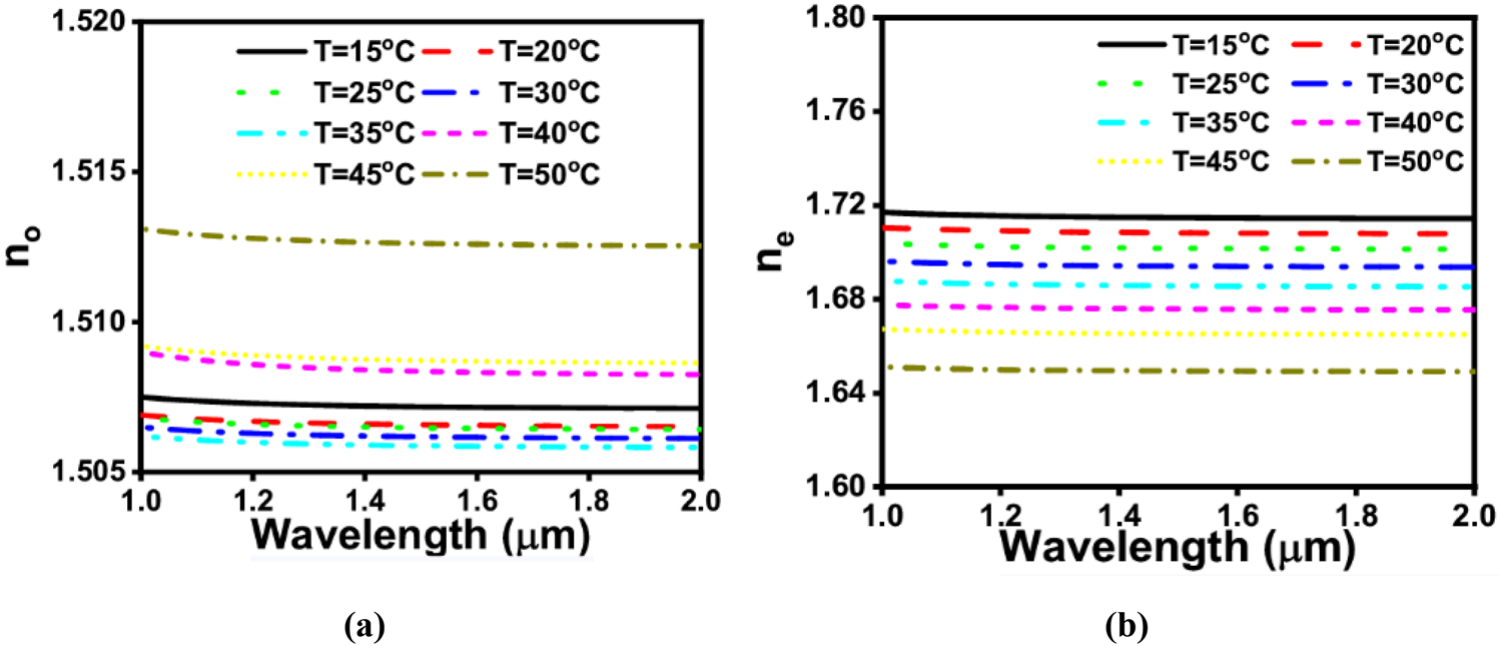
Temperature dependence of the (**a**) ordinary; n_o_ and (**b**) extraordinary; n_e_ refractive indices of the NLC material of type E7 at different temperatures from 15 to 50 °C.

The temperature dependence of n_o_ and n_e_ of the NLC of type E7 calculated from Eqs. (2) and (3)^38^ are shown in Fig. 2. It is evident from Fig. 2a that as the NLC temperature increases, n_o_ decreases in the temperature range from 15 to 35 °C. If the temperature is further increased, n_o_ will be increased. On the other hand, n_e_ decreases by increasing the NLC temperature throughout the whole temperature range from 15 to 50 °C. It is worth mentioning that the clear temperature (T_c_) of the NLC material lies between 55 and 60 °C^39^ where the LC phase will be converted to isotropic liquid phase. Thus, the operation of the proposed mode converter is limited to temperatures below 55 °C. However, to achieve mode-converting behavior in the current work, it is crucial to specify the phase-matching wavelength λ_PMW_ at each operating temperature. It is revealed from Fig. 4a,b that the phase matching points (points of intersection) between the higher-order mode in the liquid crystal core and the fundamental mode supported by the BK7 only occurs in the temperature range from 20 to 35 °C. However, no phase matching points could be attained in the temperature range from 40 to 55 °C. Thus, the proposed mode converter is limited to the temperature range 20–35 °C.

Further, the Sellmeier equation of BK7 material is taken from^35^, and is shown in Eq. (4).

4 $$n\left(\lambda \right)=\sqrt{1+\frac{{A}_{1}{\lambda }^{2}}{{\lambda }^{2}-{B}_{1}}+\frac{{A}_{2}{\lambda }^{2}}{{\lambda }^{2}-{B}_{2}}+\frac{{A}_{3}{\lambda }^{2}}{{\lambda }^{2}-{B}_{3}}}$$

where the BK7 material’s wavelength-dependent refractive index is represented by n(λ). The coefficients^35^ A_1_ = 1.03961212, A_2_ = 0.231792344, A_3_ = 1.01046945, B_1_ = 0.00600069867 μm^2^, B_2_ = 0.0200179144 μm^2^, and B_3_ = 103.560653 μm^2^. The BK7 has a good chemical stability, low density, low processing cost, and high transmission in the visible and near-infrared range^35^. In addition, as reported in^35,40^, BK7 has incredibly low optical loss at 1.55 μm (the operating wavelength of the proposed mode converter). At a temperature of 25 °C, the ordinary refractive index (n_o_) of the E7 NLC is equal to 1.5024 at λ = 1.55 μm which is very close to that of the BK7 (n_BK7_ = 1.5007). This enhances the mode conversion process where phase matching between the NLC first-order mode and the BK7 fundamental mode can be easily obtained. Thus, BK7 is chosen as the material of the left core in the proposed mode converter. It should be also noted that the refractive index of BK7 (1.5007) is lower than that of D263 glass (1.506) and Nylon 6 (1.5419), the mode will be well-confined in the BK7 core surrounded by low-index silica (1.44). The relatively thin Nylon and D263 glass layers attach a small portion of the field, while most of the light is confined in the BK7 core. Additionally, the BK7 material has extremely low confinement loss that could be neglected according to^40^.

**Figure 3:**
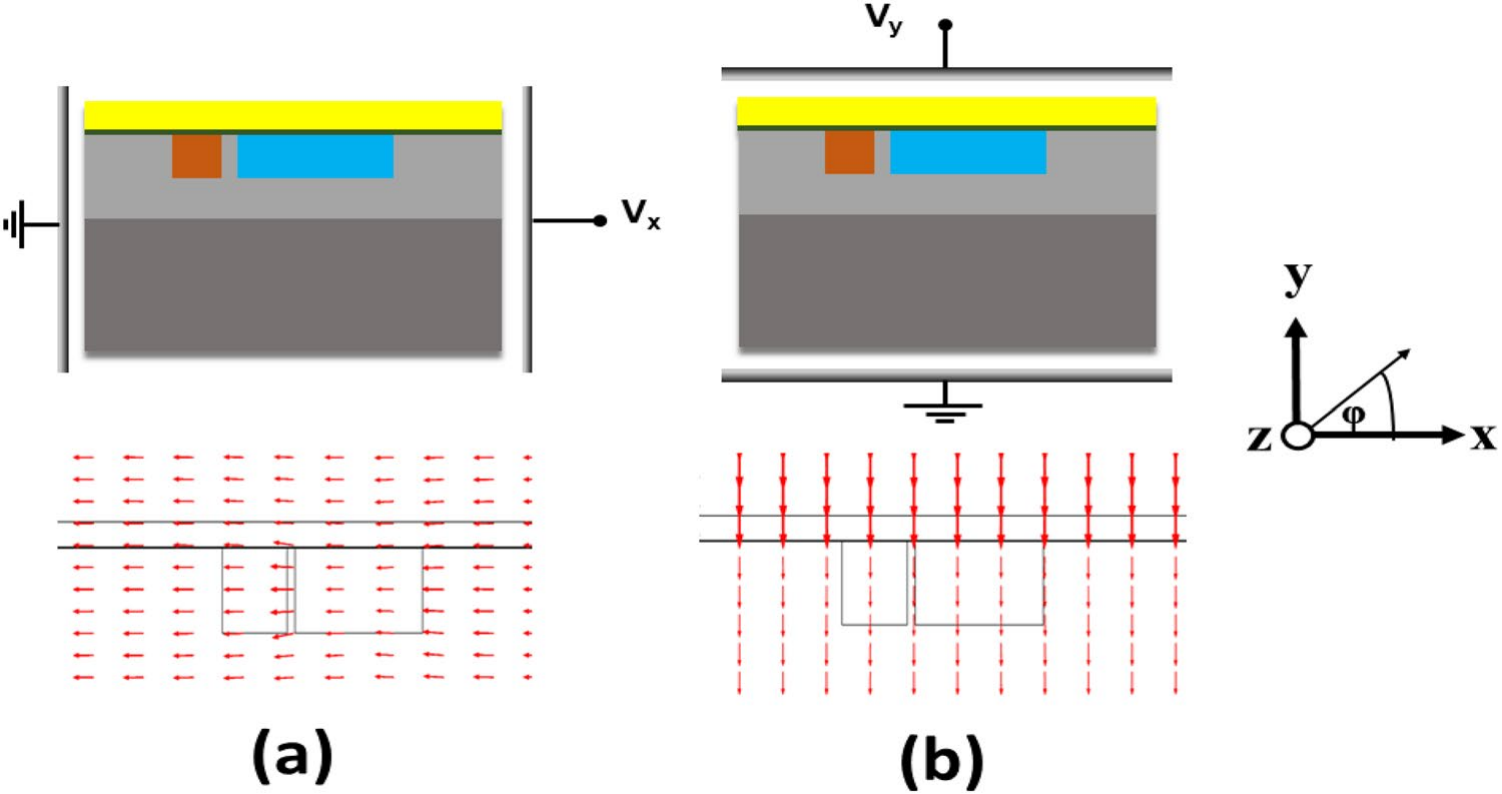
The E-field arrows inside the proposed structure while applying an external E-field between two sets of electrodes to control the orientation angle of NLC molecules at (**a**) φ = 0°, and (**b**) φ = 90°.

A non-polarized laser source is spliced to the NLC core of the proposed dual asymmetric waveguides (ADCWG) structure via a standard multi-mode optical fiber with similar dimensions to those of the NLC core^41^. Thus, the fundamental and the higher order modes supported by the NLC waveguide will be excited. The operation of the suggested mode converter depends on the coupling between the first higher-order mode supported by the NLC core and the fundamental mode of the BK7 core. This coupling can be induced at a certain wavelength thanks to the phase matching between the two modes. In the proposed structure, the NLC core has a larger width when compared to that of the BK7 core to allow the existence of higher order modes. Consequently, the fundamental mode of one of the higher order modes that the NLC core supports will excite the left core. The coupling wavelength can be controlled via the NLC temperature. Thus, a tunable mode converter can be achieved. In addition, the orientation of the NLC molecules defines the polarization of the coupled modes. As illustrated in Fig. 3, the orientation of the NLC molecules can be manipulated by applying an external electric field between two sets of vertical and horizontal electrodes. It is worth mentioning that this study is performed via COMSOL Multiphysics software^42^ using the electrostatics physics tool. The results in Fig. 3 prove the uniformity of the E-field distribution inside the NLC waveguide which ensures the uniform alignment of NLC molecules by applying the external electric field.

At λ = 1.55 μm, BK7 material has a refractive index of 1.5007^35^. Thus, the fundamental mode supported by the BK7 core would be excited by one of the higher order modes (supported by the right NLC core) that has an effective index near 1.5 i.e., n_o_. At φ = 0°, the dielectric permittivity tensor of the NLC material is given as ${\varepsilon }_{r}=[{n}_{e}^{2} {n}_{o}^{2} {n}_{o}^{2}]$. Then, through the NLC core, the y-polarized modes are affected by n_o_ while the x-polarized modes depend on n_e_. Thus, at the phase matching wavelength with the proper dimensions of the two cores, the basic y-polarized mode in the left (BK7) core is tightly linked to the first higher order y-polarized mode, provided by the NLC core. As the extraordinary refractive index i.e., n_e_ is much greater than the refractive index of BK7, no coupling occurs between the x-polarized modes in the two cores. The orientation of the NLC molecules can be rotated by applying a suitable external electric field, Fig. 3. If the electrodes are horizontally oriented, this will induce a rotation angle (φ) of 90°. In this case, the dielectric permittivity tensor of the NLC material will be changed to ${\varepsilon }_{r}=[{n}_{o}^{2} {n}_{e}^{2} {n}_{o}^{2}]$. Thus, the x-polarized modes supported by the NLC core are affected by n_o_ while the y-polarized modes of the same core sense n_e_. The first higher-order x-polarized mode, which is supported by the NLC core, is thus strongly coupled to the fundamental x-polarized mode in the BK7 core when the phase matching requirement is met. Thus, the applied electric field can be used to control the kind of linked polarization externally. The direction of the applied external electric field can be controlled by using two electrodes with an effective driving voltage of about 50 V_rms_^43^. Consequently, the rotation angle (φ) of the NLC molecules can be set according to the electric field direction. The electrodes can be integrated with SOI or SiO_2_/Si waveguides as proposed in^44^. In this context, Maune et al.^44^ have presented electrically tuned SOI based ring resonators by using the NLCs as a waveguide side and top cladding material. Moreover, Su and Hwang^45^ have studied the effect of the applied voltage on the LC orientation. When the applied voltage is less than a threshold voltage (V_th_ = 35 V), the director of the LC (RDP-98995; DIC Corp.) is barely disturbed and parallel to the fiber axis. The LCs are reoriented with a tilt angle when the applied voltage is greater than 35 V. Such a tilt angle increases with increasing the voltage gradually until the molecules become parallel to the direction of the applied electric field. Additionally, Haakestad et al.^46^ have shown that the applied voltage should be larger than 38 V_rms_ to control the NLC molecules orientation.

The BK7 material has high thermal stability as reported in^35,40^ Thus, the temperature has approximately no effect on the optical characteristics of the BK7 core. Additionally, the light propagation in the left core of the presented structure will not be affected by the external applied voltage where the BK7 material is electrically stable and has no electro-optic effects^35,47^.

## Results and discussion

The 2D simulations of the suggested ADCWG are performed to study the modal characteristics of the supported modes using the finite difference method (FDM) via the Lumerical software package^32^. In this numerical study, a mesh element size of 0.04 μm is utilized while a perfectly matched layer (PML) with a thickness of 3.0 µm is employed as a boundary condition to truncate the simulation domain. Additionally, a minimum element size of 7.2 × 10^−4^ μm is utilized with a maximum element growth rate of 1.1. Through the light propagation and crosstalk analyses, the structure is divided into 1200 divisions in the propagation direction. Initially, each core of the proposed structure is studied individually as a single waveguide. Modifying the structural properties of the two cores is necessary to attain phase matching between the targeted modes. When the initial higher order mode supported by the NLC core and the fundamental mode supported by the BK7 core have the same effective index, phase matching takes place. Due to the asymmetry of the proposed structure, phase matching will be possible only at a single wavelength known as the phase matching wavelength (λ_PMW_). In the proposed mode converter, the temperature of the NLC material can be changed to tune the λ_PMW_. Coupling length (L_c_) is defined as the propagation length at which the largest amount of power is transferred from the launching (right) core to the left core. Away from λ_PMW_, weak coupling is induced due to the absence of phase matching.

**Figure 4:**
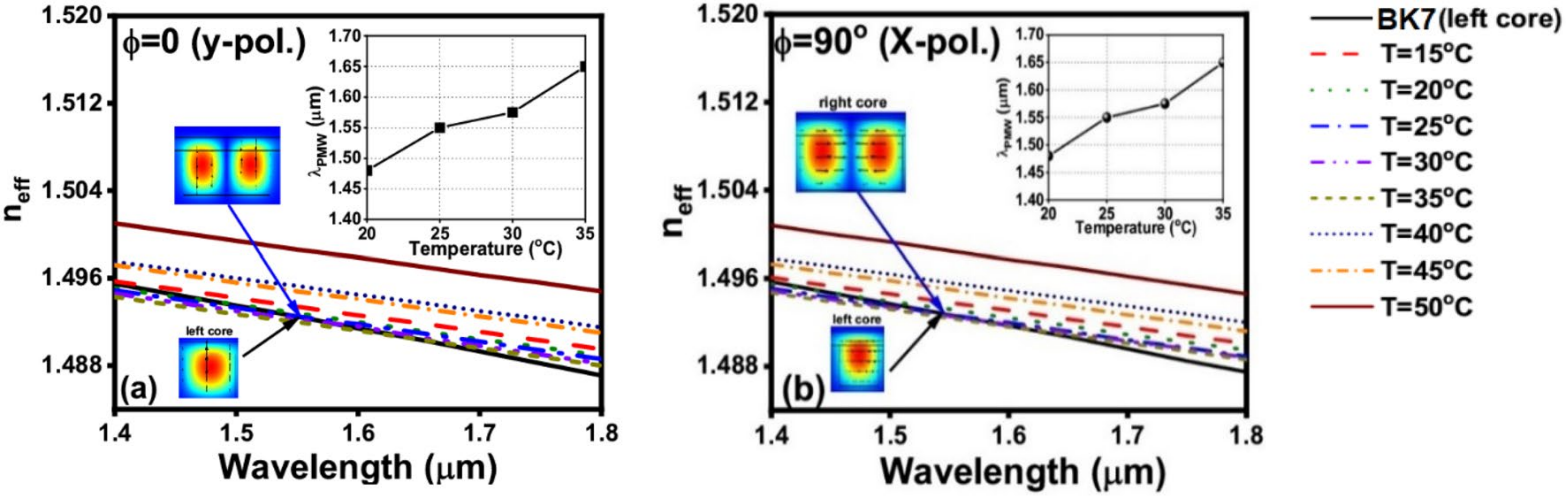
Temperature variation for practical mode index dependence on the wavelength of the fundamental mode in the left-side core and the first-order mode in the right-side core at W_R_ = 9.8 μm in both cases: (**a**) φ = 0° (y-pol.) and (**b**) φ = 90° (x-pol.).

Figure 4 depicts the effective indices (n_eff_) of the first higher order mode supported by the NLC core at different temperatures and that of the fundamental mode guided by the BK7 core. The NLC core is studied at both φ = 0° (Fig. 4a) and φ = 90° (Fig. 4b). The insets in Fig. 4a,b show the profiles of the studied modes in the two cores where the TM mode is studied at φ = 0° while the TE mode is investigated at φ = 90°. It is evident from these figures that generally, the n_eff_ of both modes decreases with the increase in wavelength. However, by increasing the NLC temperature, the n_eff_ of the studied mode decreases at φ = 0° and φ = 90°. The intersection points in both figures represent the points of phase matching between the two modes supported by the two cores. The values of λ_PMW_ increases by increasing the NLC temperature as shown in the insets of Fig. 4  a,b. Additionally, the conversion mode can be switched between the y-polarized and x-polarized modes by rotating the NLC molecules from φ = 0° to φ = 90°, respectively. Due to the temperature-dependent characteristics of the NLC material, the proposed mode converter can therefore be thermally tuned. The varied λ_PMW_ values caused by the temperature shift between 20 and 35 °C are shown in Table 1 and the insets of Fig. 4a,b. Moreover, Table 1 shows that increasing the NLC temperature from 20 to 35 °C increases λ_PMW_ from 1.483 to 1.65 μm, at φ = 0°, Fig. 4a. At φ = 90°, the λ_PMW_ increases from 1.45 to 1.616 μm by increasing the temperature from 20 to 35 °C, respectively. Additionally, as shown in Table 1 and the insets of Fig. 4a,b, even though the angle of the NLC molecules changes from φ = 0° to φ = 90° at a temperature of 25 °C, the value of λ_PMW_ remains constant at 1.55 μm. Thus, the same structure with the same dimensions can be employed for mode conversion for both TE and TM polarizations at λ = 1.55 μm with the same device length (L_D_). According to the results summarized in Table 1, the minimum temperature sensitivity, ***S***_***T***_ (Δλ_PMW_/ΔT) of the proposed mode converter is 6 nm/ °C while the maximum value is equal to 14 nm/ °C. Thus, the proposed device can be employed as a temperature sensor with quite high sensitivity.

**Table 1:** Phase matching wavelength λ_PMW_ versus temperature at both molecules’ rotation angles φ = 0° and φ = 90°, the width of the right-side core W_R_ = 9.8 μm.

Temperature (°C)	λ_PMW_ (μm) at φ = 0°	λ_PMW_ (μm) at φ = 90°
20	1.483	1.45
25	1.55	1.55
30	1.58	1.575
35	1.65	1.616

**Figure 5:**
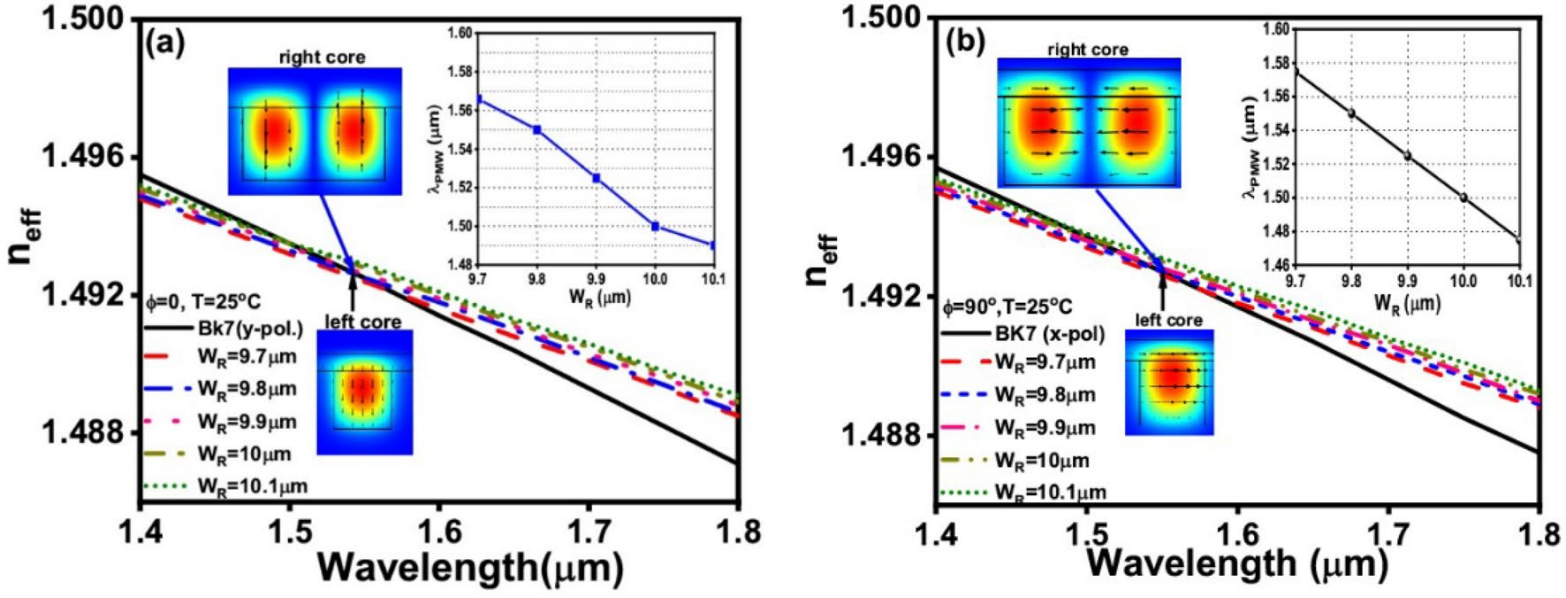
W_R_ variation for practical mode index dependence on the wavelength of the fundamental mode in the left-side core and the first-order mode in the right-side core at temperature 25 °C in both cases: (**a**) φ = 0° and (**b**) φ = 90°.

It may be easily expected that W_R_ is the most important and effective geometrical parameter in terms of the field distribution in the NLC core. Thus, it affects the n_eff_ of the modes of the right core and consequently affects the coupling process between the two cores. Therefore, a separate study on the effect of W_R_ on the n_eff_ and the λ_PMW_ is performed and the results are depicted in Fig. 5 and Table 2. It is worth mentioning that all other geometrical parameters are fixed to their initial values while the rotation angle is switched between 0° and 90°. As shown in the insets of Fig. 5a,b, λ_PMW_ decreases as W_R_ increases while T is fixed to 25 °C. Figure 5 and Table 2 confirm the stability of the λ_PMW_ at T = 25 °C and W_R_ = 9.8 μm when φ is switched from 0° to 90°. Therefore, W_R_ is fixed at 9.8 μm throughout the subsequent analysis. At φ = 0°, the y-polarized modes supported by the NLC core are affected by n_o_ while the x-polarized modes depend on n_e_. In this case, there is no coupling between the x-polarized modes in the two cores because n_e_ is significantly larger than n_BK7_. On the other hand, at φ = 90°, the y-polarized modes relies on n_e_ while the x-polarized modes supported by the NLC are affected by n_o_. It is worth noting that the NLC core is a rectangular core with W_R_ = 9.8 μm and H = 5 μm. Therefore, the y-polarized modes confinement characteristics would be different than those of the x-polarized modes as shown in the inset field plot shown in Fig. 5. Therefore, there is a slight difference between the device's behaviors when the W_R_ parameter is varied with respect to the x- polarized (φ = 90°) and y-polarized mode (φ = 0°) as shown in the inset of Fig. 5.

**Table 2:** Phase matching wavelength λ_PMW_ vs. W_R_ at φ = 0° and φ = 90°, while T = 25 °C.

W_R_ (μm)	λ_PMW_ (μm) at φ = 0°	λ_PMW_ (μm) at φ = 90°
9.7	1.5667	1.575
9.8	1.55	1.55
9.9	1.525	1.525
10	1.5	1.5
10.1	1.49	1.475

**Figure 6:**
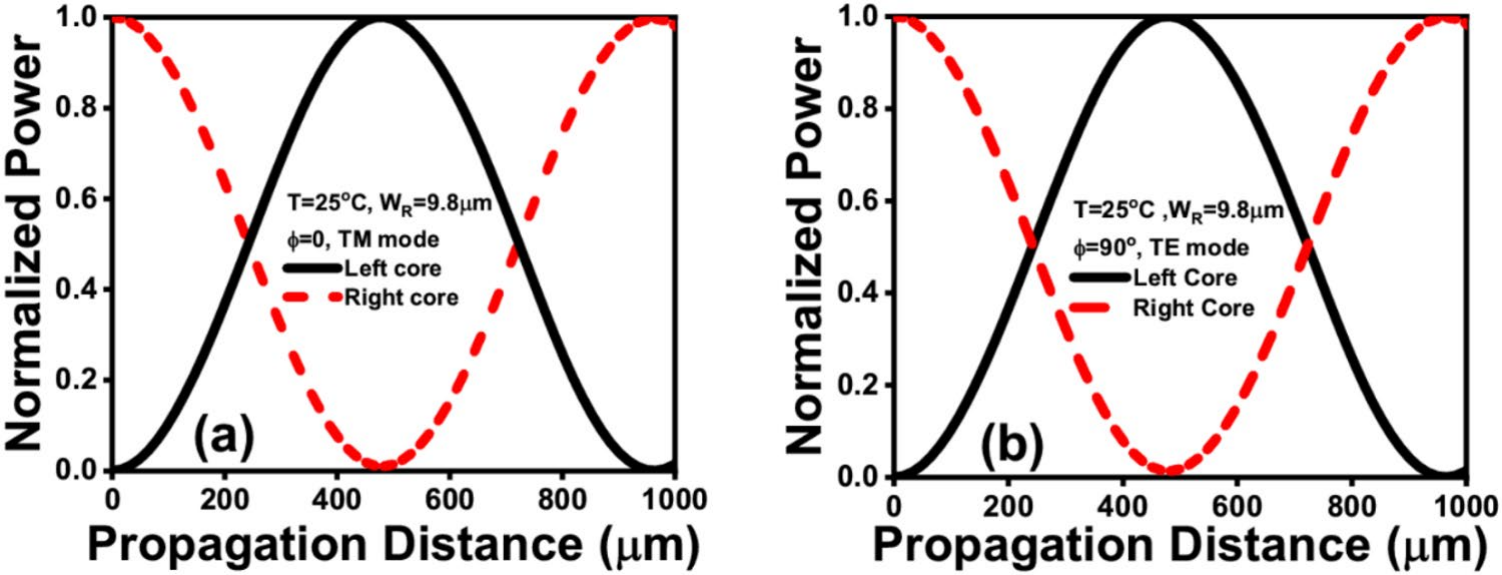
(**a**) Evolution of the normalized left-side core power for the right-side core excitation at T = 25 °C, W_R_ = 9.8 µm, and φ = 0° (TM-mode), (**b**) Evolution of the normalized left-side core power for the right-side core excitation at T = 25 °C, W_R_ = 9.8 µm, and φ = 90° (TE mode).

In order to prove the coupling concept between the two different modes supported by the two cores, a propagation study is performed. Figure 6 depicts the normalized power of the two studied modes, TE, and TM modes supported by the BK7 and the NLC cores while the rotation angle is varied between 90° and 0°, respectively. It is worth noting that this study is done by utilizing the full vectorial finite difference method (FVFDM) via the Lumerical software package^32^. Thus, the propagation characteristics of the two studied modes are calculated and the results are depicted in Fig. 6. The coupling length between any two modes can be calculated using Eq. (5)

5 $${L}_{C}=\frac{{\lambda }_{PMW}}{2\times ({n}_{eff}^{even}-{n}_{eff}^{odd})}$$

where ${n}_{eff}^{even}$ and ${n}_{eff}^{odd}$ are the effective indices of the even and odd modes supported by the proposed dual asymmetric channel’s structure.

According to Fig. 6, after a propagation distance of L_C_, almost all the normalized optical power is coupled from the NLC core to the BK7 core at λ = 1.55 μm and T = 25 °C. It is important to note that the same behavior can be attained for the two polarizations i.e., TE and TM modes by alternating the rotation angle between 0° and 90° as may be seen in Fig. 6a,b. It is significant to remember that the suggested mode converter can operate based on the TM polarization modes at φ = 0° and the TE polarization modes at φ = 90°. The normalized power coupled from the launching core to the left core reaches its highest value at a propagation distance of 482.24 μm at φ = 0°. Conversely, at φ = 90°, the maximum normalized power coupled from the right core achieves its highest value after a propagation distance of 482.384 μm. The two propagation distances are quite different, so, an average propagation length of (482.24 + 482.384)/2 = 482.31 μm is considered.

**Figure 7:**
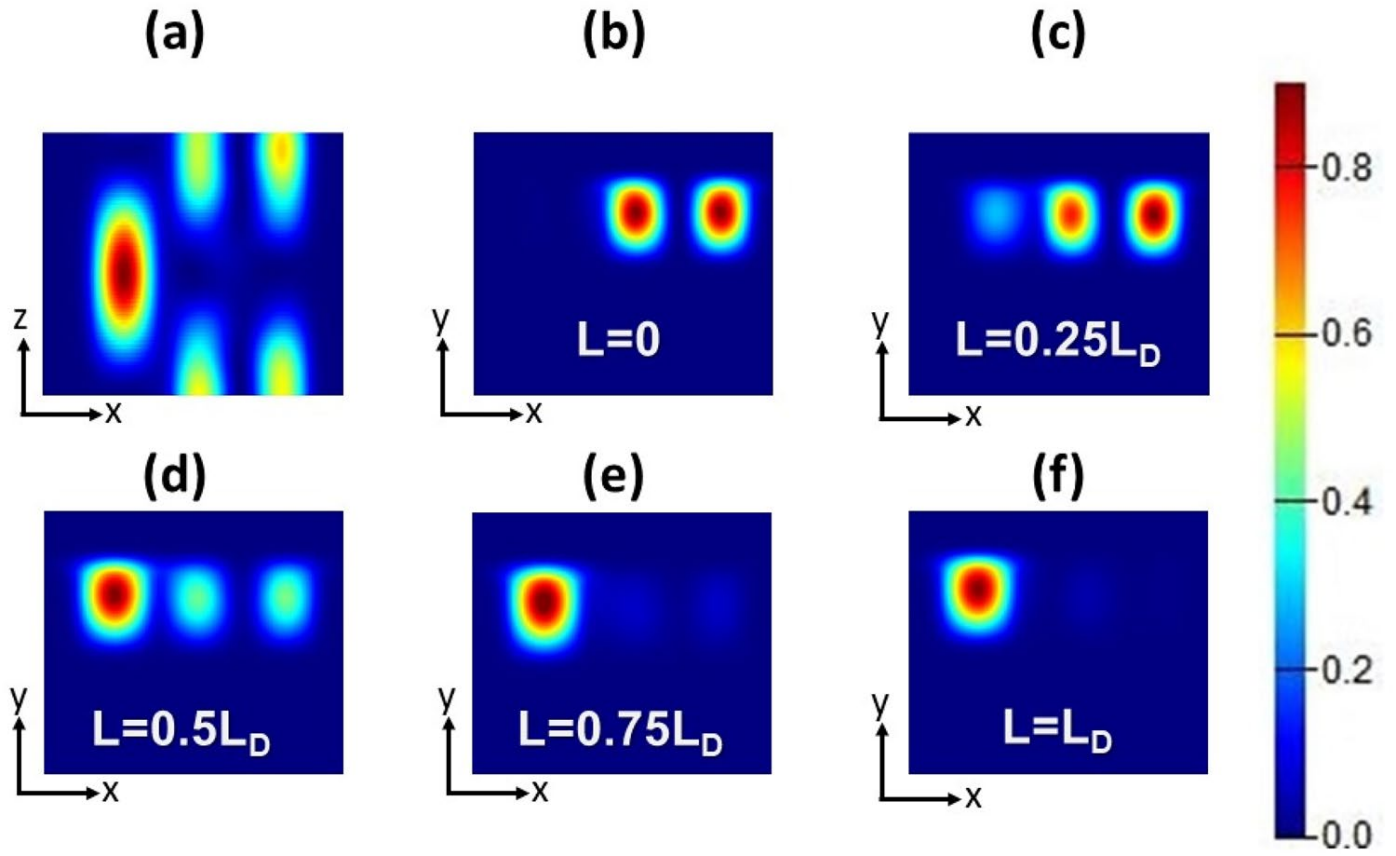
(**a**) x–z view of the propagation electric field through the proposed ADCWG. E-field distribution in the dual-core structure at (**b**) z = 0, (**c**) z = 0.25L_D_, (**d**) z = 0.5L_D_, (**e**) z = 0.75L_D_, and (**f**) z = L_D_ with a fixed operating wavelength of 1.55 µm, NLC temperature 25 °C, and rotation angle φ = 90°.

**Figure 8:**
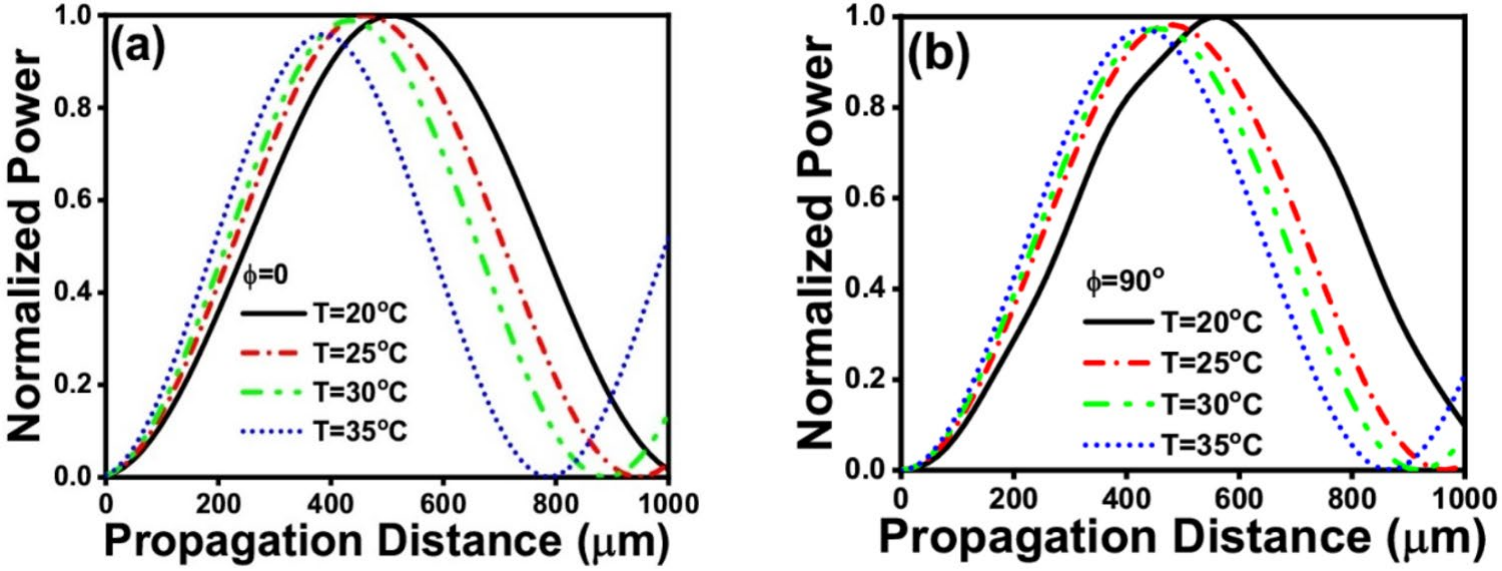
Normalized powers of the mode in the left-side core at different temperatures at: (**a**) φ = 0° and (**b**) φ = 90°.

To prove the concept of power coupling between the two modes supported by the two cores, a propagation study is carried out via Lumerical software package^32^. It is worth noting that this study is performed with the optimum geometrical parameters where φ is taken as 90° and T = 25 °C. Figure 7 shows the evolution of the E-field in the dual cores at various propagation distances. As may be seen from Fig. 7a, the power coupling between the two cores occurs alternatively and a maximum power transfer from the NLC core to the BK7 core occurs at L_D_ = 482.31 μm. Figure 7b–f depict the field profiles at different cross sections along the propagation direction starting from the excitation plane (Fig. 7b) passing through intermediate distances (Fig. 7c–e) and ending with the field profile at exactly a distance of L_D_ (Fig. 7f). It is revealed from Fig. 8 that at L_D_ = 482.31 μm, most of the power is coupled from the launching core to the other one at λ = 1.55 μm.

The evolution of the normalized power in the BK7 core is shown in Fig. 8 for both φ = 0° and φ = 90°, at various temperatures. This study is carried out to prove the temperature tunability of the proposed higher-to-fundamental mode converter. As may be seen in Fig. 8, by changing the NLC temperature, approximately the same amount of power is coupled to the fundamental mode supported by the left (BK7) core. However, the coupling length differs slightly from one temperature to another. So, an average device length of 482.31 μm is considered to keep the power coupling maximum at T = 25 °C and as high as possible for all other studied temperatures.

Table 3 summarizes the calculated coupling lengths at different studied temperatures for φ = 0° and φ = 90°. It is revealed by the results summarized in Table 3 that L_C_ decreases with the increase in temperature. The average L_C_ at T = 25 °C is selected as the ultimate device length. This may affect the efficiency of mode conversion at temperatures away from 25 °C. However, the overall performance is acceptable through the whole studied temperature range i.e., 20–35 °C.

**Table 3:** Effect of temperature’s variation on the coupling length (L_c_).

Temperature (°C)	L_C_ (μm) at φ = 0°	L_C_ (μm) at φ = 90°	L_C_ (μm) (average)
20	530.23	553.78	542.01
25	482.24	482.384	482.31
30	451.67	455.03	453.35
35	402	433.74	417.87

**Figure 9:**
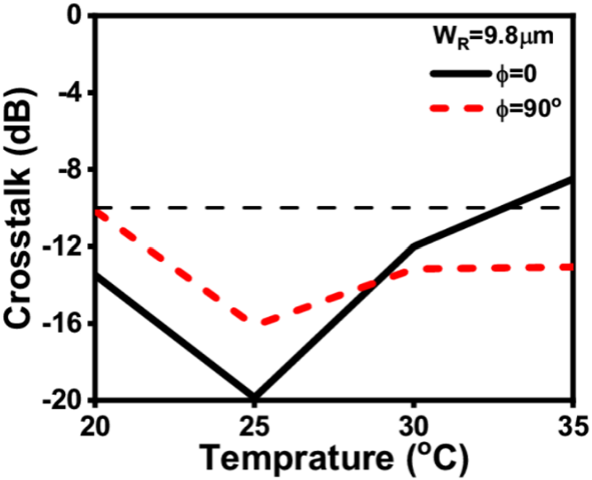
Effect of the temperature on the CT of the proposed device.

In order to show the performance of the proposed mode converter at different temperatures, the crosstalk parameter is calculated. The crosstalk (CT) can be obtained from Eq. (6) as reported in^48^:

6 $$CT=10{log}_{10}\left(\frac{{P}_{undesired}}{{P}_{desired}}\right)$$

where P_undesired_ is the power fraction remains in the launching core exactly after a distance of L_D_ while P_desired_ is the power fraction coupled from the launching core to the BK7 core at L_D_. Using Eq. (6), the proposed ADCWG is found to give a minimum CT of − 19.86 dB at T = 25 °C, φ = 0°, and 16.1 dB at T = 25 °C, φ = 90°, at the optimum geometrical parameters as may be seen in Fig. 9. Further, the CT is still better than − 10 dB over the temperature range from 20 to 35 °C.

A tolerance analysis for the geometrical parameters of the proposed design is also studied to show its robustness to expected fabrication errors. The effect of the geometrical parameters (W_R_, W_L_, H, t and d) are tested. It is worth mentioning that at each step, one parameter is varied while all other parameters are fixed to their optimum values. Table 4 summarizes the effects of the different geometrical parameters on λ_PMW_, L_C_, and CT. The obtained results reveal that decreasing W_R_ by 2% and 4% from its optimum value (9.8 µm) induces an increase to the values of λ_PMW_, a reduction to the L_C_, and an increase in the CT. On the other hand, increasing W_R_ by + 2% and + 4% decreases λ_PMW_, increases L_C_, and decreases CT. However, changing W_L_ by ± 2% or ± 4% has a slight effect on λ_PMW_ and CT while the change in L_C_ is quite larger as may be verified by the results in Table 3. Additionally, decreasing H by 2% and 4% from its optimum value (5 µm) has a slight effect on λ_PMW_, L_C_, and CT. It may be also seen from Table 4 that the Nylon 6, and glass layer have approximately no effect on the operation of the proposed mode converter in terms of λ_PMW_, L_c_, and CT. It is worth mentioning that the silicon material is used as a substrate to support the structure due to fabrication considerations. Such a layer is located away from the core regions due to the large depth of the silica cladding. So, the thickness of the silicon substrate has no effect on the operation of the proposed mode converter.

**Table 4:** The obtained λ_PMW,_ coupling length L_C_, and crosstalk due to the fabrication tolerance considering variations in W_R_, W_L_, H, t and d.

Parameter	Tolerance (%)	λ_PMW_ (μm)	L_C_ (μm)	CT (dB)
W_R_ (optimum value = 9.8 μm)	W_R_-4%	1.65	401.5	− 9.07
	W_R_-2%	1.583	449.7	− 14.64
	W_R_ = 9.8 μm	1.55	482.2	− 19.86
	W_R_ + 2%	1.499	531.1	− 14.76
	W_R_ + 4%	1.45	582	− 12.51
W_L_ (optimum value = 5 μm)	W_L_-4%	1.525	471.9	− 19.28
	W_L_-2%	1.55	469.1	− 19.28
	W_L_ = 5 μm	1.55	482.2	− 19.86
	W_L_ + 2%	1.6	454.7	− 17.27
	W_L_ + 4%	1.65	436	− 15.06
H (optimum value = 5 μm)	H-4%	1.583	453	− 17.27
	H-2%	1.583	453.5	− 17.27
	H = 5 μm	1.55	482.2	− 19.86
	H + 2%	1.599	449.3	− 17.35
	H + 4%	1.575	466.3	− 17.21
t (optimum value = 50 nm)	t-4%	1.55	482.2	− 19.94
	t-2%	1.55	482.2	− 19.88
	t = 50 nm	1.55	482.2	− 19.86
	t + 2%	1.55	482.1	− 19.82
	t + 4%	1.55	481.9	− 19.73
d (optimum value = 1.5 μm)	d-4%	1.533	488.4	− 18.95
	d-2%	1.533	487.3	− 19.41
	d = 1.5 μm	1.55	482.2	− 19.86
	d + 2%	1.55	481	− 19.78
	d + 4%	1.55	468.9	− 19.37

In order to show the superiority of the presented design to those reported in literature with similar function^28,29,49–52^, a comprehensive comparison is performed and summarized in Table 5. In most of the included papers in this comparison, the reported converters are based on PCF structures so, it suffers from complex fabrication processes when compared to the presented simple structure in our work. Notably, this comparison takes into account the device length, operating wavelength, and tunability capability. Most published mode converters^28,29,49,51^ show non tunable behavior and large device lengths that are usually longer than the proposed device, as clarified in Table 5. Moreover, the proposed mode converter has a higher ***S***_*T*_ than those reported in^50,52^ and a wider wavelength range than those summarized in Table 5. Consequently, the proposed converter operates more efficiently than the other mode converters that have been reported previously in literature.

**Table 5:** Comparing the outcomes of the suggested ADCWG mode converter to those reported in the literature.

References	Structure	Tunability	λ_PMW_ (μm)	L_D_ (μm)	*S*_*T*_ (nm/°C)
^28^	Mode converter based on mode coupling in an ADC-PCF	No	1.55	12,700	–
^29^	Hybrid dual-core PCF	No	1.55	3210	–
^49^	Mode converter based on dual-core all-solid PCF	No	1.55	6430	–
^50^	Mode converter based on polymer waveguide grating	Yes	1.56–1.592	5070	–
^51^	Adiabatically tapered MOF mode converter	No	1.55	21,000	–
^52^	Mode converter based on the long-period fiber gratings written in the six-mode fiber	Yes	1.5–1.54	24,000	–
Present work	ADCWG mode converter	Yes	1.483–1.65	482.31	14

## Conclusion

The investigation and numerical analysis of a compact, tunable mode converter based on an ADCWG with an NLC core are presented in this paper. The power coupling between the first order mode of the NLC core and fundamental mode of the BK7 core are studied at a temperature range from 20 to 35 °C, showing a temperature tunability of the mode conversion process. According to the numerical results, the NLC temperature can be used to regulate the coupling wavelength over the range of 1.45 to 1.65 μm. Additionally, the fabrication tolerance of the reported design is investigated. At λ = 1.55 μm, a compact mode converter with L_D_ of 482.31 μm and a good CT of − 19.86 dB is attained.

## Data Availability

The datasets used and/or analyzed during the current study available from the corresponding author on reasonable request.

## References

[1] Baum C, Jaffe M, Palm L, Kumar A, Simon J (2023). Optical mode conversion via spatiotemporally modulated atomic susceptibility. Opt. Express.

[2] Chen G, Zhang R, Sun J (2015). On-chip optical mode conversion based on dynamic grating in photonic-phononic hybrid waveguide. Sci. Rep..

[3] Pires DG, Rocha JCA, Jesus-Silva AJ, Fonseca EJS (2019). Optical mode conversion through nonlinear two-wave mixing. Phys. Rev. A.

[4] Nakazawa M (2014). Exabit optical communication explored using 3M scheme. Jpn. J. Appl. Phys..

[5] Van Uden RGH (2014). Ultra-high-density spatial division multiplexing with a few-mode multicore fibre. Nat. Photonics.

[6] Celler GK, Cristoloveanu S (2003). Frontiers of silicon-on-insulator. J. Appl. Phys..

[7] Liu S (2022). Thermo-optic phase shifters based on silicon-on-insulator platform: State-of-the-art and a review. Front. Optoelectron..

[8] Marshall A, Natarajan S (2007). SOI Design: Analog, Memory and Digital Techniques.

[9] Colinge J-P (2004). Silicon-on-Insulator Technology: Materials to VLSI.

[10] Steglich, P. & You, K. Y. Silicon-on-insulator slot waveguides: Theory and applications in electro-optics and optical sensing. *Emerg. Waveguide Technol.* 187–210 (2018).

[11] Tan Q, Huang X, Zhou W, Yang K (2013). A plasmonic based ultracompact polarization beam splitter on silicon-on-insulator waveguides. Sci. Rep..

[12] Dai D (2018). 10-Channel Mode (de) multiplexer with dual polarizations. Laser Photonics Rev..

[13] Cheng Z (2019). Sub-wavelength grating assisted mode order converter on the SOI substrate. Opt. Express.

[14] Jimenez-Durango, C. *et al.* A novel interferometric sensor based on a dual-core transversally chirped microstructured optical fiber for measuring glucose concentration. In *2018 International Conference on Electromagnetics in Advanced Applications (ICEAA)* 531–534 (IEEE, 2018).

[15] Markos C, Yuan W, Vlachos K, Town GE, Bang O (2011). Label-free biosensing with high sensitivity in dual-core microstructured polymer optical fibers. Opt. Express.

[16] Eggleton BJ, Kerbage C, Westbrook PS, Windeler RS, Hale A (2001). Microstructured optical fiber devices. Opt. Express.

[17] Bise RT (2002). Tunable photonic band gap fiber. Optical Fiber Communication Conference ThK3.

[18] Hameed MFO, Obayya SSA, Wiltshire RJ (2010). Beam propagation analysis of polarization rotation in soft glass nematic liquid crystal photonic crystal fibers. IEEE Photonics Technol. Lett..

[19] Younis BM, Heikal AM, Hameed MFO, Obayya SSA (2018). Highly wavelength-selective asymmetric dual-core liquid photonic crystal fiber polarization splitter. JOSA B.

[20] Hameed MFO, Obayya SSA (2010). Analysis of polarization rotator based on nematic liquid crystal photonic crystal fiber. J. Lightwave Technol..

[21] Obayya S, Hameed MFO, Areed NFF (2016). Computational Liquid Crystal Photonics: Fundamentals, Modelling and Applications.

[22] Saitoh K, Florous NJ, Varshney SK, Koshiba M (2008). Tunable photonic crystal fiber couplers with a thermo-responsive liquid crystal resonator. J. Lightwave Technol..

[23] Chen HL (2014). A novel polarization splitter based on dual-core photonic crystal fiber with a liquid crystal modulation core. IEEE Photonics J..

[24] Khan KR, Bidnyk S, Hall TJ (2012). Tunable all optical switch implemented in a liquid crystal filled dual-core photonic crystal fiber. Prog. Electromagn. Res. M.

[25] Khalil AE (2018). Highly sensitive photonic crystal fiber biosensor based on titanium nitride. Opt. Quantum Electron..

[26] Montoya Cardona JA, Gomez Cardona ND, Gonzalez Valencia E, Torres Trujillo P, ReyesVera E (2019). Tunable mode converter device based on photonic crystal fiber with a thermo-responsive liquid crystal core. Photonics.

[27] Lin G, Dong X (2012). Design of broadband LP 01↔ LP 02 mode converter based on special dual-core fiber for dispersion compensation. Appl. Opt..

[28] Chen M-Y, Zhou J (2008). Mode converter based on mode coupling in an asymmetric dual-core photonic crystal fibre. J. Opt. A Pure Appl. Opt..

[29] Cai S (2015). Hybrid dual-core photonic crystal fiber for spatial mode conversion. IEEE Photonics Technol. Lett..

[30] Yu Y, Sun B (2018). Ultra-wide-bandwidth tunable magnetic fluid-filled hybrid connected dual-core photonic crystal fiber mode converter. Crystals.

[31] Mahmoud MMH, Younis BM, Areed NFF, Hameed MFO, Obayya SSA (2021). Tunable liquid crystal asymmetric dual-core photonic crystal fiber mode converter. Appl. Opt..

[32] https://www.lumerical.com/.

[33] https://scipoly.com/technical-library/refractive-index-of-polymers-by-index/

[34] Hameed MFO, Hussain FFK, Obayya SSA (2017). Ultracompact polarization rotator based on liquid crystal channel on silicon. J. Lightwave Technol..

[35] SCHOTT Zemax catalog 2017-01-20b (obtained from http://www.schott.com).

[36] Tan CZ (1998). Determination of refractive index of silica glass for infrared wavelengths by IR spectroscopy. J. Non. Cryst. Solids.

[37] Hameed MFO, Obayya SSA, Al-Begain K, el Maaty MIA, Nasr AM (2009). Modal properties of an index guiding nematic liquid crystal based photonic crystal fiber. J. Lightwave Technol..

[38] Li J, Wu S-T, Brugioni S, Meucci R, Faetti S (2005). Infrared refractive indices of liquid crystals. J. Appl. Phys..

[39] Brugioni S, Meucci R (2007). Refractive indices of the nematic mixture E7 at 1550 nm. Infrared Phys. Technol..

[40] Eaton SM (2008). Low-loss waveguides fabricated in BK7 glass by high repetition rate femtosecond fiber laser. Appl. Opt..

[41] https://www.thorlabs.com/

[42] COMSOL Multiphysics, https://www.comsol.com

[43] Wei L, Alkeskjold TT, Bjarklev A (2009). Compact design of an electrically tunable and rotatable polarizer based on a liquid crystal photonic bandgap fiber. IEEE Photonics Technol. Lett..

[44] Maune B, Lawson R, Gunn C, Scherer A, Dalton L (2003). Electrically tunable ring resonators incorporating nematic liquid crystals as cladding layers. Appl. Phys. Lett..

[45] Su H-P, Hwang S-J (2023). A novel approach to fabricate high performance electrically tunable fiber device based on well-aligned liquid crystal-infiltrated hollow core fiber. Opt. Laser Technol..

[46] Haakestad MW (2005). Electrically tunable photonic bandgap guidance in a liquid-crystal-filled photonic crystal fiber. IEEE Photonics Technol. Lett..

[47] Hassanein GN (2019). Optimizing low frequency electro-optic response of nematic liquid crystals. Optik (Stuttg).

[48] Hameed MFO, Esmail MSM, Obayya SSA (2020). Terahertz photonic crystal fiber polarization rotator. JOSA B.

[49] Zhang Y (2015). Mode converter based on dual-core all-solid photonic bandgap fiber. Photonics Res..

[50] Yang Y, Chen K, Jin W, Chiang KS (2015). Widely wavelength-tunable mode converter based on polymer waveguide grating. IEEE Photonics Technol. Lett..

[51] Taher AB (2016). Adiabatically tapered microstructured mode converter for selective excitation of the fundamental mode in a few mode fiber. Opt. Express.

[52] Zhao, X. *et al.* Mode converter based on the long-period fiber gratings written in the six-mode fiber. In *2017 16th International Conference on Optical Communications and Networks* (*ICOCN*) 1–3 (IEEE, 2017).

[53] Chenming, H. U. Losses of a nematic liquid-crystal optical waveguide (1974).

[54] Green M, Madden SJ (1989). Low loss nematic liquid crystal cored fiber waveguides. Appl. Opt..

[55] Dhawan A, Canva M, Vo-Dinh T (2011). Narrow groove plasmonic nano-gratings for surface plasmon resonance sensing. Opt. Express.

[56] Bozhevolnyi SI, Volkov VS, Devaux E, Laluet J-Y, Ebbesen TW (2006). Channel plasmon subwavelength waveguide components including interferometers and ring resonators. Nature.

[57] d’Alessandro A, Bellini B, Donisi D, Beccherelli R, Asquini R (2006). Nematic liquid crystal optical channel waveguides on silicon. IEEE J. Quantum Electron..

[58] Beccherelli R, Manolis IG, d’Alessandro A (2005). Characterisation of photoalignment materials for photonic applications at visible and infrared wavelengths. Mol. Cryst. Liq. Cryst..

[59] Xu Y, Uddin MA, Chung PS, Chan HP (2009). Polymer planar waveguide device using inverted channel structure with upper liquid crystal cladding. Opt. Express.

[60] d’Alessandro A, Martini L, Gilardi G, Beccherelli R, Asquini R (2015). Polarization-independent nematic liquid crystal waveguides for optofluidic applications. IEEE Photonics Technol. Lett..

[61] d’Alessandro A, Asquini R (2021). Light propagation in confined nematic liquid crystals and device applications. Appl. Sci..

[62] Xing Y (2015). Digitally controlled phase shifter using an SOI slot waveguide with liquid crystal infiltration. IEEE Photonics Technol. Lett..

